# Pancreatic Tuberculosis with Vascular Involvement and Peritoneal Dissemination in a Young Man

**DOI:** 10.1155/2017/4396759

**Published:** 2017-09-07

**Authors:** Meng Zhu, Ning Zhang, Wei Tao, Zhitao Wang, Shuixiang He

**Affiliations:** ^1^Department of Gastroenterology, Affiliated Hospital of Xi'an Jiaotong University, 277 West Yanta Road, Xi'an, Shaanxi, China; ^2^Department of Pathology, General Hospital of Ningxia Medical University, Ningxia, China; ^3^Department of Gastroenterology, General Hospital of Ningxia Medical University, Ningxia, China; ^4^Department of Radiology, General Hospital of Ningxia Medical University, Ningxia, China

## Abstract

Pancreatic tuberculosis (TB) is an extremely rare form of extrapulmonary tuberculosis even in endemic areas that masquerades as a mass or inflammation because of lack of typical clinical manifestations and radiologic features and therefore usually misdiagnosed as a pancreatic malignancy or pancreatitis. Here we present a 23-year-old young man with pancreatic tuberculosis mimicking pancreatic head carcinoma A man who suffered from upper abdominal pain and nausea for half a month was admitted to our hospital. Narrow band imaging (NBI) and gastroscopic imaging, together with endoscopic ultrasonography (EUS), revealed a duodenal bulb mucous prominences lesion. Computed tomography (CT) and magnetic resonance imaging (MRI) both suggested a pancreatic mass which resembled a pancreatic head tumor that had a higher risk of malignancy. The patient therefore accepted an exploratory laparotomy and pancreatoduodenectomy, Whipple operation. Biopsies of pancreas, duodenum, lymph nodes, omentum, and adipose tissues were all performed, revealing tuberculosis infection in pancreas, hepatic portal vein infiltration, and peritoneal dissemination. The patient was treated successfully after operation and recovered with standard anti-TB drugs for 6 months. Timely reporting of this rare case can help physicians improve their ability to identify several specific illnesses and diseases that share confusing signs or symptoms clinically and radiographically.

## 1. Introduction

Tuberculosis (TB) is a common infectious disease and remains a major global public hygiene problem and social problem especially in developing countries such as South Africa and Asia. It not only typically affects lungs (pulmonary TB) but can also affect other organs (extrapulmonary TB) [1]. Approximately one-eighth of pulmonary TB patients have extrapulmonary involvement, of which abdominal cavity might be affected with the possibility of 11%. In order of descending frequency, the locations of abdominal involvement are the colon, jejunum, appendix, duodenum, stomach, esophagus, sigmoid colon, and rectum [2]. Pancreatic/parapancreatic TB is an uncommon form of abdominal infection and has a rare occurrence even in endemic regions. It accounts for less than 5% of all tuberculosis in the developing world [3]. Many epidemiological analyses suggest that the probability of tuberculosis is much higher among human immunodeficiency virus- (HIV-) positive people. On average, 15% of TB patients will have positive result for HIV testing [1]. Tuberculosis in abdomen includes pancreatic TB, because it usually mimics an unresectable malignant tumor clinically and radiologically and therefore many cases can get the diagnosis only after histopathological examination of the specimens obtained from Whipple's surgery [4, 5]. We provide a case report here of a patient who underwent surgery with the preoperative diagnosis of pancreatic carcinoma but thoroughly changed to benign tuberculosis after surgical biopsies. We also discuss the occurrence of pancreatic TB, diagnosis, and pathological process, thereby highlighting the importance of understanding of rare disease that shares similar signs or characteristics to other common ailments in abdominal cavity.

## 2. Case Report

In March 2016, a 23-year-old young man was admitted to our hospital with main complaint of abdominal pain and nausea after diet as well as loss of appetite for half a month. The vague abdominal pain or discomfort was predominantly in the epigastrium. Meanwhile, the concomitant symptoms did not include fever, night sweat, reflux, heartburn, vomiting, haematemesis, and melena. Physical examination was unremarkable except for mild tenderness over the upper abdomen. The patient's medical history pointed out that he did not have any special diseases and had not been previously diagnosed as thoracic TB or other organ's TB. Other than that, it should be noted that the patient had lost 6 pounds of weight just only half a month.

**Figure 1:**
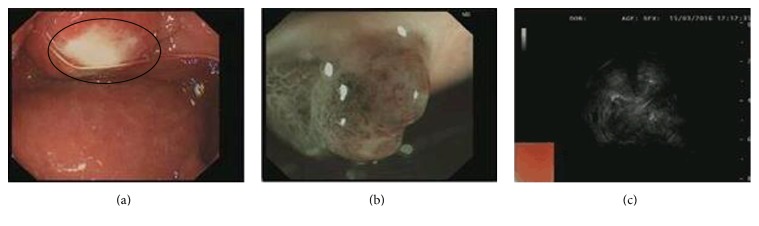
Electronic gastroscope and endoscopic ultrasonography both showed the abnormality of the duodenal mucosa. (a) Electronic gastroscope revealed that there was a mucosal protrusion at the first portion of the duodenum (duodenal bulb) with an ulcerated surface (marked with a circle). (b) NBI and ME showed that the local vascular was disordered, and gland duct disappeared. (c) EUS showed that the hypoechoic mass with uniform echo was at duodenal bulb.

**Figure 2:**
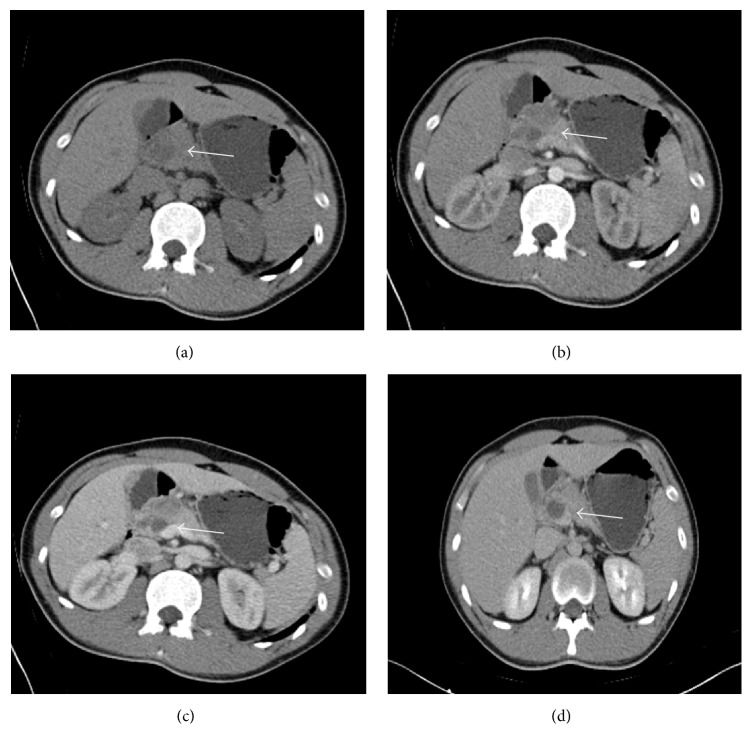
The CT scan revealed that the enlargement of the head of pancreas had an irregular density and unclear boundary. Moreover, inhomogeneous enhancement and progressive enhancement appeared after using iodinated contrast material. (a) CT scan, (b) arterial phase, (c) portal phase, and (d) delayed phase image.

**Figure 3:**
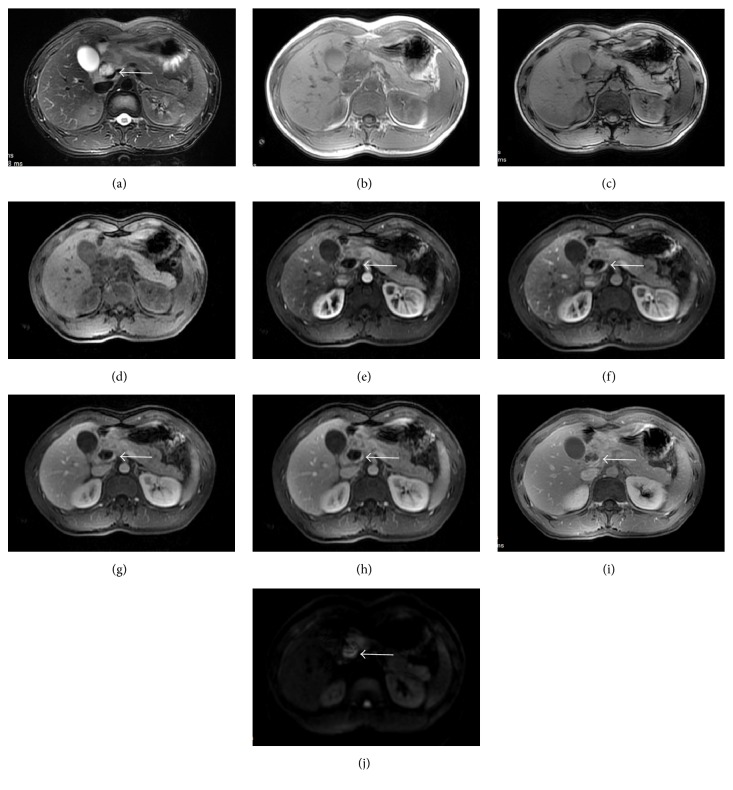
The MRI graph revealed that the head of the pancreas increased with abnormal signal, T2W1 showed a mixed signal, mainly with high signal, T1W1 showed a low signal, positive and negative phase showed no lipid component within the lesion, and DWI showed a high signal which was delayed enhancement unevenly. In addition, there were a slight dilatation of common bile duct and no dilatation of pancreatic duct. T2W1 graph (a), positive and negative phase diagrams (b-c), LAVA prescanned graph (d), LAVA graphs (e–h), delayed enhancement graph (i), and DWI graph (j).

The blood routine test and the function of liver and kidneys were all within normal range. The serological tests for HBV, HCV, HIV, and syphilis were all negative. Carcinoembryonic antigen (CEA) and cancer antigen 19-9 (CA19-9) were both not elevated. Electronic gastroscope showed that there was a mucosal protrusion at duodenal bulb with an ulcerated surface in the center, coated with whitish fur. Narrow band imaging (NBI) and magnifying endoscope (ME) showed that the local vascular was disordered and gland duct disappeared. Endoscopic ultrasonography (EUS) showed that the duodenal bulb contained hypoechoic mass on the top of the raised center. The echo inside was uniform but the duodenal wall lacked unity and coherence. The lesion was closely adjacent to the serosa (Figure 1). The gastroscope biopsy displayed an inflammation in duodenal mucosa and low-grade atypical hyperplasia in some glands (data not shown). Computed tomography (CT) showed an irregular low density lesion in the neck of the pancreas, with unclear margins of size 3.7 × 4.5 cm that enhanced after iodinated contrast material. Hepatic hilar region and intrahepatic cholangiectasis were both found. CT images also revealed that the arteria hepatica was enveloped which mimicked a pancreatic head carcinoma (Figure 2). Magnetic resonance imaging (MRI) revealed enlarged pancreatic head and neck, long T1/T2 signal, and high DW1 signal. Furthermore, common bile duct was constricted by pancreatic mass, resulting in dilated bile duct of liver inside and outside. The contrast enhanced MRI had heterogeneous density, with the vascular involvement; therefore, the neoplastic changes should be strongly considered (Figure 3). Besides these, we also give the patient the chest X-ray to approve or eliminate the possibility of primary tuberculosis. The result showed that there was no abnormality found in chest X-ray (data not shown).

**Figure 4:**
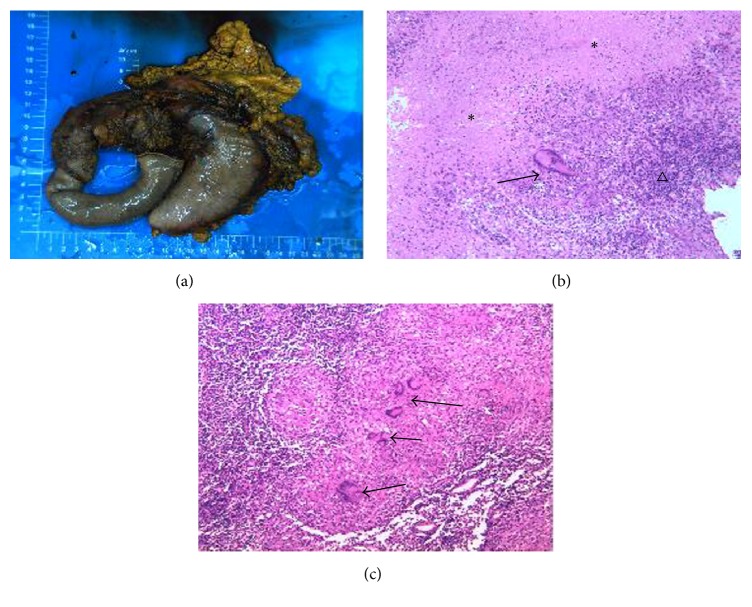
The histopathological pictures. (a) Whipple surgical specimens contained the pancreas, duodenum, partial stomach, and the omentum. The lesion was located on the pancreatic head. ((b) and (c)) HE staining. Granulomatous nodules consisting of lymphocytes, epithelioid cells, and multinucleated giant cells can be seen at the site of the lesion. Necrosis can be seen in the middle of nodules which were marked with an asterisk. The triangle marked the lymphocytes around the nodules. The arrow indicated the multinucleated giant cells reaction. These pathological features suggested tuberculous lesions.

According to the illness history and plenty of examinations above, although the vascular involvement supported the diagnosis of malignant tumor, other examinations did not show any surgical contraindication, and the patient was a young man, and his families had strong treatment preferences. To clarify the disease, only through surgery can doctors obtain the tissues to do pathological diagnosis. For this reason, we ultimately gave the patient exploratory laparotomy under general anesthesia. Intraoperative findings were as follows: the lesion located in the pancreatic head, 6 × 5 × 3 cm in size, which had an obscure boundary and hard texture. The gallbladder was not large, choledochectasia and the portal vein were invaded, and multiple enlarged lymph nodes around pancreas and the fifth groups of stomach were seen and touched. Therefore, the pancreas and duodenum were resected using Whipple procedure (pancreatoduodenectomy). Histopathologic specimens returned as follows: (1) the granuloma, which was comprised of lymphocyte, epithelioid cell, and polykaryocyte, could be seen in the multiple nodules and fibrous adipose tissues between the junctions of the pancreatic head and duodenum. The caseous necrosis appeared frequently in the center of several granulomas. These features are diagnostic criteria for tubercular nodules. (2) The portal vein was composed of smooth muscle, in which lymphocyte and a little number of polykaryocytes infiltrated. (3) Tubercular nodules were seen in the lymph nodes of intestinal adipose tissues. (4) Individual caseating granulomatous inflammation and necrosis were found in the omentum of lesser curvature of stomach. (5) Many lymph nodes isolated from operation displayed the pathological characteristics of tuberculosis (Figure 4). The patient was thereafter advised to transfer to the Intensive Care Unit to accept the administration of standardized anti-TB medical drugs including 300 mg/d isoniazid, 450 mg/d rifampicin, 1500 mg/d pyrazinamide, and 750 mg/d ethambutol for 6 months. The symptoms relieved and the patient recovered progressively after that. At present, one year after surgery, the patient is doing well with no adverse effects from surgery and takes part in work and study normally.

## 3. Discussion

Tuberculosis, a time-honored disease, still has a high morbidity nowadays and ranks as the leading cause of death for infectious disease worldwide according to the World Health Organization (WHO) [6]. As known, tuberculosis always occurs in respiratory system such as lung which is the most predominant loci. The gastrointestinal tract is the sixth most predilection site for extrapulmonary tuberculosis, and the incidence will even be higher when the patients were infected with HIV virus [7]. Often mycobacterium tuberculosis* (Mtb)* infects extrapulmonary organs such as gastrointestinal tract through deglutition or blood-borne infection. Under this susceptible circumstance, gastrointestinal tract exposes itself to infection with* Mtb*; however, the organs are usually intestine, colon, and abdominal cavity. The pancreas, as an organ in abdomen, could rarely be invaded by tubercle bacillus, although pancreatic TB had already been described early in 1944 [8]. The pancreatic head is primarily affected in the process of pancreatic TB occurrence [3].

A spectrum of manifestations during the development of pancreatic TB is really diverse, ranging from abdominal discomfort, abdominal pain, nausea and vomiting, obstructive jaundice, loss of appetite, and weight loss to fever, night sweats, or abdominal mass [9–11]. Although there are so many symptoms for TB, patient always afflicts one or more kinds of presentations, as is demonstrated here. Our patient presented with nonspecific epigastric pain and weight loss in a short time. Based upon these, some neoplastic disease cannot be excluded although the patient was not old. When the pancreatic carcinoma or neoplasm occurred, the most common symptoms are turned to pain, weight loss, jaundice, and anorexia, which will become more and more serious [8, 9]. In our case, we found that the malignancy should be highly suspected because the radiology defined exactly that a mass had low density, compressed the surrounding organs, and wrapped the blood vessels. The surgical operation also supported that pancreatic mass invaded the portal vein and infiltrated multiple lymph nodes which means peritoneal dissemination. Postoperative pathology, however, had given a startling reversal. Pancreatic TB replaced pancreatic cancer as the final diagnosis.

A correct preoperative diagnosis of pancreatic TB is difficult. Our patient achieved valuable information from imaging about the size and nature of the lesions; however, the likelihood of malignant tumor was emphasized more. It is because there are no distinctive phenotypes on imaging that can distinguish tubercular lesions from pancreatic carcinoma [12]. We consider that the reason why the misdiagnosis cannot be avoided may be because the abdominal organs are the most dense area of the body, the density is similar, the organs are overlapped and covered, and fluid and gas within the abdominal cavity as well as gastrointestinal motility could all influence the judgment. Moreover, our patient's pancreatic TB accompanied with peritoneal dissemination and vascular invasion made it exactly hard to separate from malignant tumor at primary diagnosis.

Vascular invasion, which is usually considered an inoperable malignancy and described as a point of differentiating benign lesions from malignant lesions in most cases, could also appear in pancreatic TB and cannot be used as a standard to discriminate between pancreatic TB and malignancy. Rana et al. [13] reported that patients who diagnosed pancreatic TB after cytopathological confirmation had imaging features of vascular invasion by the pancreatic head mass. Both arterial and venous involvement can be observed. Peritoneal dissemination, usually a bad outcome of advanced tumor, may become a form of manifestations during the progression of abdominal tuberculosis. We report the case that has peritoneal dissemination expressed as multiple enlarged lymph nodes within the cavity. Coincidentally, Sousa MD reported that tuberculosis bore a striking resemblance to malignancy so that a hypothesis of cancer with peritoneal dissemination had been accepted before histopathological analysis showed a tubercular disease [14].

EUS has an excellent performance on obtaining high resolution images by a closely placed transducer. However, none of the EUS features of pancreatic TB are distinctive. Therefore, cytological examination originating from EUS-guided fine needle aspiration (EUS-FNA) is mandatory to differentiate between benign and malignant mass lesion because material obtained from the lesions can be immediately sent for pathology which is recognized as the gold-standard for preoperative diagnosis [15]. It is widely accepted that patients with lymphatic diseases (such as tuberculosis) or solid tumor (such as pancreatic carcinoma) should undergo EUS-FNA whose tissue-based diagnose is far better that others [16]. Outcomes of EUS-FNA results, however, vary from endoscopist's experience to cytopathologist's skills. It is regrettable that the FNA technology in our unit has not yet been introduced, but it is fortunate for the patient because both of the intraoperation and postoperation found that the tuberculosis had developed a large number of intra-abdominal disseminations which enabled laparotomy to be a necessary option to achieve a better prognosis than conservative treatment.

Pathological confirmation is indispensable for establishing the ultimate diagnosis of tuberculosis. The pathologic feature of tuberculosis is a granulomatous inflammation with signs of necrosis. Tuberculosis caused by* Mtb* is the most common disease in the formation of caseous granuloma [17]. Granuloma, the central part, is caseous necrosis in which mycobacteria DNA can be found with numbers ranging from 0 to 9% [18], the peripheral part is clusters of immune cells including macrophages, epithelioid cells, multinucleated giant cells, Langerhans cells, and lymphocyte [19]. For a strict diagnosis, the pathological sections of our patient both took on characteristics of caseous necrosis and infiltration of numerous inflammatory cells.

Why pancreatic TB is rare? It is not easy for tubercular pathogens to survive and progress in human pancreas. It is attributed to the resistance provided by the pancreatic enzymes. Pancreatic enzymes, including lipases and deoxyribonucleases, have antimycobacterial effects to interfere with the seeding of* Mtb *[20]. That is the reason why pancreatic TB usually occurs in immunocompromised individuals or as a complication of miliary TB. The pathogenic steps of* Mtb *are as follows. First,* Mtb* must be able to adhere to host cells, penetrate, and persist in host cells. Second,* Mtb *must be able to avoid, evade, or compromise the host defense response. Third,* Mtb* must be able to release virulent factors to damage the host tissue or organs, such as sulfatides, cord factors, and wax D, components of the cell wall. Last,* Mtb* must be able to exist in one host and become highly contagious [21]. With regard to our case, several reasons are considered: (1) some occult* Mtb* within the abdomen being reactive when the body resistance is lower, through hematogenous or direct spread of abdominal lymph node, (2) dysfunction of pancreatic exocrinosity, (3) excessive immune response, and (4) ingestion of food contaminated by* Mtb* with very destructive virulent factors.

In conclusion, pancreatic TB is an infrequent extrapulmonary site with unremarkable presentations and insidious images that delay its diagnosis and treatment. The presence of vascular invasion and peritoneal dissemination cannot exclude possibility of benign pancreatic diseases like tuberculosis. Final diagnosis relies on pathology. A combination of surgery and antituberculotics could cure pancreatic TB.
